# High *PTEN* gene expression is a negative prognostic marker in human primary breast cancers with preserved p53 function

**DOI:** 10.1007/s10549-017-4160-5

**Published:** 2017-02-17

**Authors:** Synnøve Yndestad, Eilin Austreid, Stian Knappskog, Ranjan Chrisanthar, Peer Kåre Lilleng, Per Eystein Lønning, Hans Petter Eikesdal

**Affiliations:** 1 Section of Oncology, Department of Clinical Science, University of Bergen, Bergen, Norway; 2 Department of Oncology, Haukeland University Hospital, Bergen, Norway; 3 Department of Pathology, Haukeland University Hospital, Bergen, Norway; 4 The Gade Laboratory for Pathology, Department of Clinical Medicine, University of Bergen, Bergen, Norway; 5 Section of Molecular Pathology, Department of Pathology, Oslo University Hospital, Oslo, Norway

**Keywords:** Locally advanced breast cancer, PTEN, p53, Prognosis, Predictive factors

## Abstract

**Purpose:**

PTEN is an important tumor suppressor in breast cancer. Here, we examined the prognostic and predictive value of *PTEN* and *PTEN pseudogene (PTENP1)* gene expression in patients with locally advanced breast cancer given neoadjuvant chemotherapy.

**Methods:**

The association between pretreatment *PTEN* and *PTENP1* gene expression, response to neoadjuvant chemotherapy, and recurrence-free and disease-specific survival was assessed in 364 patients with locally advanced breast cancer given doxorubicin, 5-fluorouracil/mitomycin, or epirubicin versus paclitaxel in three phase II prospective studies. Further, protein expression of PTEN or phosphorylated Akt, S6 kinase, and 4EBP1 was assessed in a subgroup of 187 tumors.

**Results:**

Neither *PTEN* nor *PTENP1* gene expression level predicted response to any of the chemotherapy regimens tested (*n* = 317). Among patients without distant metastases (*n* = 282), a high pretreatment *PTEN* mRNA level was associated with inferior relapse-free (RFS; *p* = 0.001) and disease-specific survival (DSS; *p* = 0.003). Notably, this association was limited to patients harboring *TP53* wild-type tumors (RFS; *p* = 0.003, DSS; *p* = 0.009). *PTEN* mRNA correlated significantly with *PTENP1* mRNA levels (*r*_s_ = 0.456, *p* < 0.0001) and PTEN protein staining (*r*_s_ = 0.163, *p* = 0.036). However, no correlation between PTEN, phosphorylated Akt, S6 kinase or 4EBP1 protein staining, and survival was recorded. Similarly, no correlation between *PTENP1* gene expression and survival outcome was observed.

**Conclusion:**

High intratumoral *PTEN* gene expression was associated with poor prognosis in patients with locally advanced breast cancers harboring wild-type *TP53*.

**Electronic supplementary material:**

The online version of this article (doi:10.1007/s10549-017-4160-5) contains supplementary material, which is available to authorized users.

## Introduction

Mutations in the *TP53* tumor suppressor gene, encoding the p53 protein, are associated with lack of response to anthracycline- and mitomycin-containing chemotherapy as well as poor prognosis in breast cancer [1–7]. However, some patients experience lack of response to these chemotherapeutic compounds despite a preserved tumor p53 function, pointing to additional resistance mechanisms [8]. Apart from p53, PTEN is an important tumor suppressor which is frequently inactivated in breast cancer, thus enabling increased signaling of the crucial growth-promoting PI3K-Akt-mTOR pathway [9, 10]. PI3K-Akt-mTOR signaling is involved in resistance to endocrine- and HER2-directed therapy clinically [9, 11], as well as resistance to chemotherapy in preclinical trials [12, 13]. This suggests that PTEN expression may influence response to cancer treatment.

While *PTEN* somatic mutations are rare, PTEN protein expression is frequently lost in breast carcinomas, pointing to transcriptional and post-transcriptional regulation as possible mechanisms [14, 15]. Of notice, PTEN and p53 reciprocally interact to preserve each other’s protein levels [16]. Further, in vitro data from prostate cancer cell lines suggest that *PTEN pseudogene (PTENP1*) mRNA transcripts may regulate the *PTEN* expression level by competing for *PTEN*-degrading micro RNAs (miRNAs) [17].

The aim of the present study was to assess the prognostic role of pretreatment *PTEN* and *PTENP1* gene expression levels in patients with locally advanced breast cancer, stratified by *TP53* mutations status, and the predictive role of *PTEN* and *PTENP1* gene expression levels toward chemotherapy response. In addition, we examined protein expression levels of PTEN as well as key signaling molecules in the PI3K-Akt-mTOR pathway [9]. For this purpose, we used tumor material collected from patients with locally advanced breast cancer treated with different chemotherapy regimens in phase II trials conducted between 1991 and 2007 [1–5].

## Methods

### Patient material

**Fig. 1:**
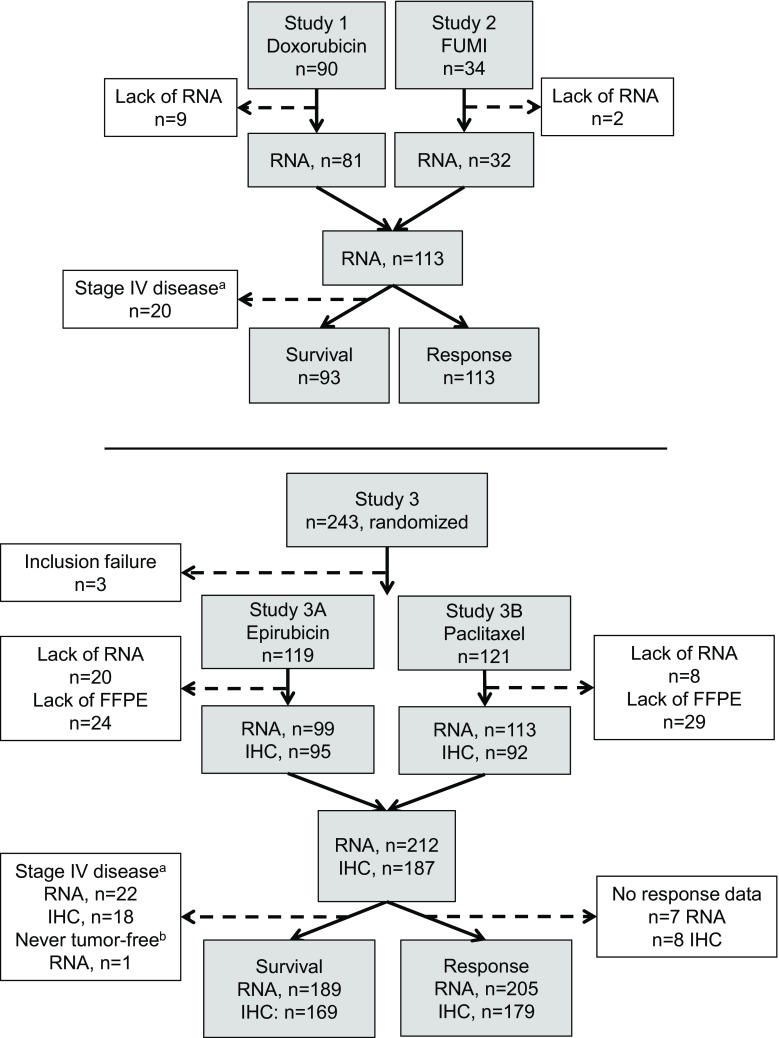
Flow chart depicting the number of patients with locally advanced breast cancer recruited in Studies 1–3, and the number of samples available from each trial for RNA and immunohistochemistry (IHC) analysis. In Study 3, patients randomized to either epirubicin or paclitaxel were switched to the opposite regimen if tumor regression on the first regimen was insufficient; survival analysis was performed for all patients randomized to each regimen (intention-to-treat) and separately for those patients without crossover (w/o cross) to the opposite regimen. ^a^Patients with stage IV disease were excluded from survival analysis. ^b^One patient with progressive disease (PD) never became tumor-free, and recurrence-free or disease-free survival could therefore not be assessed. *FFPE* formalin-fixed paraffin-embedded tissue, *IHC* immunohistochemistry

Pretreatment tumor samples were available from patients with locally advanced breast cancer (T3/T4 and/or N2/N3) included in three neoadjuvant phase II trials described in detail previously [1, 3–5, 18] and outlined in Fig. 1. Dates of enrollment of the first participants to the trials were 18/1-91 (Study 1), 1/6-93 (Study 2), and 24/11-97 (Study 3). In Study 1, patients were given neoadjuvant doxorubicin, 14 mg/m^2^ qW for 16 weeks. In Study 2, each patient received 5-fluorouracil 1000 mg/m^2^ and mitomycin 6 mg/m^2^ (FUMI) q3w for 12 weeks. In Study 3, patients were randomized to either epirubicin 90 mg/m^2^ (Arm A) or paclitaxel 200 mg/m^2^ q3w (Arm B), administered in 4–6 courses. Further, in Study 3, patients with suboptimal tumor response to either drug switched to the opposite chemotherapy regimen [5, 18].

Response rates (according to the The Union for International Cancer Control criteria), TNM status, estrogen receptor (ER), and *TP53* mutation data have been reported previously [1, 5, 18], and are summarized in Table 1, along with the current assessment of *PIK3CA* and HER2 status. Follow-up data were available for >10 years or up to time of death for all patients in the trials. A total of 317 patients were assessed for chemotherapy response with respect to gene and protein expression. Among these, 282 patients with stage 3 disease at diagnosis were used for survival analysis.

**Table 1:** Baseline patient and tumor characteristics

Treatment	Study 1^a^	Study 2^a^	Study 3A^b^	Study 3B^b^
	*Doxorubicin*	*FUMI*	*Epirubicin*	*Paclitaxel*
Patients	90	34	119	121
Accrual	1991–1997	1993–2001	1997–2003	1997–2003
Age (years)				
Range	32–88	37–82	28–70	25–70
Median	64	67	49	48
T stage				
T2^c^	3	2	1	1
T3	54	15	99	90
T4	33	17	18	30
N stage				
N0^d^	30	9	52	45
N1	34	14	48	59
N2	26	11	17	17
N3	0	0	1	0
M stage				
M0	78	24	109	106
M1	12	10	10	15
ER				
Negative	13^e^	11^e^	52	49
Positive	77	23	66	69
Unknown	0	0	1	3
HER2				
Negative^f^	24	27	63	66
Positive	6	6	30	28
Unknown	60	1	26	27
*TP53*				
*TP53* wt^g^	64	16	84	89
*TP53* mut.	26	18	23	25
Unknown	0	0	12	7
Response^h^				
PD	5	9	10	14
SD	45	13	49	47
PR	31	10	56	47
CR	0	0	4	5
Unknown	0	0	0	8
TMA^i^				
Stage 3	0	0	88	81
Stage 4	0	0	7	11
RNA/DNA^j^				
Stage 3	71	22	90	99
Stage 4	10	10	9	14
*PTEN* ^k^				
*PTEN* wt	0	0	80	99
*PTEN* mut.	0	0	2	2
Unknown	0	0	27	4
*PIK3CA* ^l^				
*PIK3CA* wt	26	20	82	92
*PIK3CA* mut.	4	12	25	22
Unknown	51	0	12	7

^a^Data from Studies 1–2 were pooled for statistical analysis due to a low number of patients in Study 2

^b^Data from Study 3 were split into Study 3a (epirubicin) and 3b (paclitaxel), based on the primary chemotherapy given

^c^T2 tumors only included if axilla stage N2. T stage and all subsequent tumor characteristics given for stage 3 and 4 combined

^d^N stage by clinical assessment alone

^e^ER negative if tumor ER concentration <10 fmol/mg in Study 1–2. ER assessed by standard IHC in Study 3

^f^For Studies 1–2; HER2 assessment available from a subset of the tumors by in situ hybridization only. For Study 3: HercepTest IHC was performed on all tumors, and HER2 in situ hybridization for tumors with staining score 2 by IHC

^g^ *TP53* mutation status, whole exome assessed by Sanger sequencing. *wt* wild-type, *mut* mutation

^h^Progressive disease (PD), stable disease (SD), partial response (PR), complete response (CR)

^i^Subset of patients from whom formalin-fixed paraffin-embedded (FFPE) tumor tissue was available for protein analysis to correlate against gene expression results (*PTEN*), response rates (stage 3 and 4 disease), or survival (stage 3 only)

^j^Subset of patients from whom tumor RNA was available for gene expression analysis to correlate against response rates (stage 3 and 4 disease) or survival (stage 3 only)

^k^Subset of patients from whom tumor DNA was available for *PTEN* mutation analysis

^l^Subset of patients from whom tumor DNA was available for *PIK3CA* mutation analysis to correlate against response rates (stage 3 and 4 disease) or survival (stage 3 only)

### Tumor samples

In each protocol, tumor samples were collected by incisional biopsies prior to commencing cancer therapy. Samples were snap frozen and stored in liquid nitrogen until DNA/RNA analysis. In the present investigation, tumor RNA was available from 325 patients; 81 patients from Study 1, 32 patients from Study 2, and 212 patients from Study 3. Among patients with tumor RNA available, seven lacked response data and 43 had primary metastatic disease, leaving 318 patients for response evaluation and 282 patients for survival analysis with respect to gene expression results (Fig. 1).

Pretreatment formalin-fixed paraffin-embedded (FFPE) tumor tissue was available from 193 patients in Study 3 as tissue microarrays (TMAs), but due to the lack of tumor tissue in some core biopsies or staining artifacts, incl. missing cores, only 187 patients could be evaluated for any particular protein. Among patients with TMA tumor tissue available, seven lacked response data, 18 had primary metastatic disease, whereas one patient did not undergo breast surgery and was unfit for calculation of recurrence-free survival, leaving 179 patients for response evaluation and 169 patients for survival analysis with respect to protein staining results (Fig. 1).

### Basic genomic procedures

Procedures, primers, and antibodies used for RNA and DNA analysis are described in detail in Online Resource 1.

### Immunohistochemistry (IHC) and in situ hybridization (ISH)

Procedures used for IHC and ISH analysis are described in detail in Online Resource 1. The antibodies used for protein analysis were monoclonal anti-Akt (phosphorylated Ser 473), monoclonal anti-HER2 (4B5, Dako), polyclonal anti-PTEN, polyclonal anti-S6 kinase (S6K, phosphorylated Ser 371, Abcam), mouse monoclonal anti-S6K (phosphorylated Thr 389), and polyclonal anti-4EBP1 (phosphorylated Thr 70). All antibodies were developed in rabbit, and purchased from Cell Signaling unless specified otherwise. Immunostaining was evaluated by two independent researchers, and given a semi-quantitative score of 0 (no staining) to 3 (strong staining). Whereas both nuclear and cytoplasmic staining were assessed for PTEN, cytoplasmic staining was scored for 4EBP1, and nuclear staining for Akt and S6K. In a combined PI3K pathway analysis, absent PTEN protein staining, phosphorylated Akt staining, phosphorylated S6K staining, and *PIK3CA* mutation were each given a score of one each, and “PI3K pathway activation” was defined as a score of two or higher.

### Statistics

Correlation analysis between *PTEN* mRNA expression level and PTEN staining was performed using Spearman's rho. Mann–Whitney test was used for comparison of mRNA or protein staining levels between tumor subgroups. The Chi-square test was used to assess the correlations between *PIK3CA* mutation status and phosphorylation status of Akt, S6 K, 4EBP1 proteins or between *PIK3CA* mutations and response to chemotherapy. Chi-square test was also used to assess the correlation between IHC staining and chemotherapy response. Survival data were assessed by Cox regression analysis calculating hazard ratios for each parameter. For Kaplan–Meier plots, patient subgroups were compared by the log-rank test. Due to a smaller number of patients, the survival data from Studies 1 to 2 were analyzed in concert, as described previously [1]. Recurrence-free (RFS) and disease-specific survival (DSS) were defined as time from inclusion in the trial until breast cancer recurrence or death due to breast cancer, respectively. Deaths for reasons other than breast cancer, or patients still alive at the time of analysis, were treated as censored observations. *PTEN* and *PTENP1* gene expression values were sorted for each of the three trials separately and divided by the median value into two groups defined as *PTEN* or *PTENP1* “low” (i.e., below the median) and “high” (i.e., above the median). Multivariate analysis was performed using Cox regression to evaluate the independent prognostic impact of *PTEN, PTENP1, TP53, PIK3CA*, HER2, and ER status in this cohort of locally advanced breast cancers. Statistical analyses were performed using the SPSS 22/PASW 17.0 and Graph Pad Prism v6 software packages. All p-values reported are two-tailed, and *p* < 0.05 was considered statistically significant.

## Results

### *PTEN, PTENP1, and TP53* gene expression

**Fig. 2:**
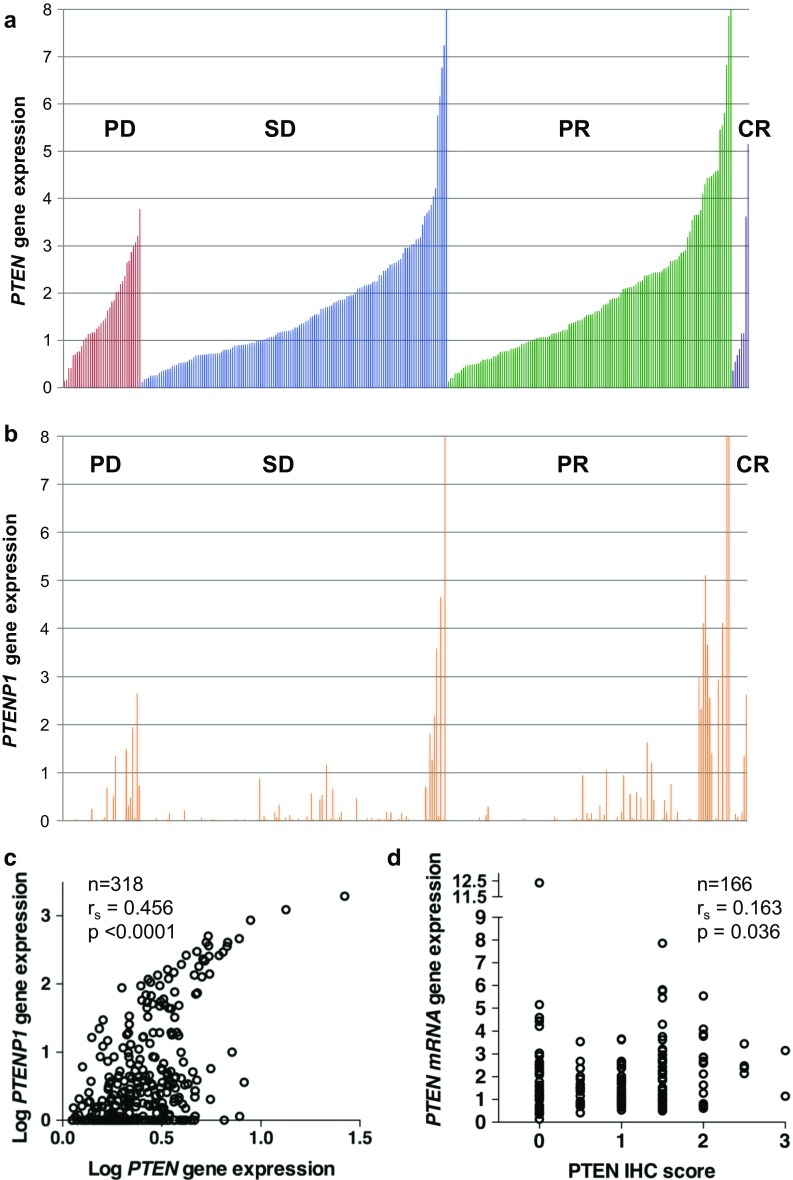
**a** Gene expression of *PTEN* in locally advanced human breast cancers prior to starting neoadjuvant epirubicin, paclitaxel, doxorubicin, or 5-FU/mitomycin (FUMI), Studies 1–3 combined. Sorted by response group and increasing *PTEN* levels. **b** Gene expression of *PTEN pseudogene (PTENP1)* in locally advanced human breast cancers prior to starting neoadjuvant chemotherapy, sorted by response group and increasing *PTEN* levels (same as **a**). **c** Scatter plot depicting the correlation between *PTEN* and *PTENP1* gene expression in breast cancers from the epirubicin/paclitaxel, doxorubicin, FUMI trials combined. **d** Scatter plot depicting the correlation between *PTEN* gene expression and PTEN protein expression in breast cancers from the epirubicin/paclitaxel, doxorubicin, FUMI trials combined. *PTEN* and *PTENP1* mRNA levels in **a**–**d** are depicted as the mean gene expression of three separate real-time RT-PCR runs, as a fraction of *RPLP2* expression, and corrected for cDNA pool. Gene expression in **a**–**b** is not depicted beyond eight times the *RPLP2* expression to visualize better differences between the tumor samples. *PD* progressive disease, *SD* stable disease, *PR* partial response, *CR* complete response

Baseline patient and breast cancer characteristics from Studies 1-3 are summarized in Table 1. *PTEN* gene expression by quantitative/real-time PCR (qPCR) was detectable in all 318 tumors with a defined treatment response (Fig. 2a). In contrast, *PTENP1* expression was undetectable in 96 tumors (30%; Fig. 2b). There was a significant, albeit not uniform correlation between *PTEN* and *PTENP1* mRNA expression levels (*r*_s_ = 0.456, *p* < 0.0001; Fig. 2c). Whereas *PTEN* mutations were identified in four out of 183 breast cancers (2.2%), *PIK3CA* mutations were found in 63 out of 220 (29%), and *TP53* mutations in 92 out of 253 (36%) tumors analyzed (Table 1). Among the four tumors with *PTEN* mutations, two had *PTEN* gene expression above and two below the median (data not shown). No significant differences in *PTEN* or *PTENP1* gene expression were observed in subgroups stratified by ER, HER2, *PIK3CA*, or *TP53* mutation status or by comparison of triple-negative breast cancer (ER/PGR/HER2 negative; TNBC) vs. non-TNBC (data not shown). *TP53* gene expression was undetectable in seven out of 273 tumors (2.5%), and a significant correlation was observed between *TP53* and *PTEN* gene expression in these 273 tumors from Studies 1 to 3 where both transcripts were measured (*r*_s_ = 0.227, *p* < 0.0002). This correlation between *TP53* and *PTEN* mRNA levels remained significant (*r*_s_ = 0.150, *p* < 0.05), if 47 out 212 tumors with known *TP53* or *PTEN* mutations (Study 3) were excluded from the analysis.

### PTEN and PI3K pathway protein expression

IHC staining results for PTEN, and phosphorylated Akt (Ser 473), S6K (Ser 371 or Thr 389), and 4EBP1 (Thr 70) are summarized in Online Resource 2. High-quality immunostaining was observed for all antibodies used, apart from phosphorylated S6K (Thr 389) which yielded poor staining of the tissue microarrays. At the same time, it has been established previously that phosphorylation at the S6K Ser371 phosphorylation site is essential for Thr389 phosphorylation [19], indicating that the staining results for Ser371 should correlate to Thr389 staining. A weak correlation (*r*_s_ = 0.163, *p* = 0.036) was established between *PTEN* gene expression and the corresponding PTEN protein staining level in 166 tumors from which both RNA and TMA tissue blocks were available (Fig. 2d). However, there was no correlation between a low *PTEN* gene expression level and increased Akt (Ser 473) or S6K (Ser 371 or Thr 389) phosphorylation in breast cancers from which both RNA and IHC tissue samples were available for such comparisons (*n* = 163). Also, there was no correlation between the absence of PTEN protein staining and increased Akt (Ser 473) or S6K (Ser 371 or Thr 389) phosphorylation by comparison of IHC tissue samples (data not shown). “PI3K pathway activation,” defined as two or more of the following: absent PTEN staining, phosphorylated Akt, phosphorylated S6K, and/or *PIK3CA* mutations, was observed in 117 out of 159 breast cancers in Study 3. *PTEN* gene expression was significantly higher (*p* = 0.028) in tumors with pathway activation, compared to tumors without pathway activation (data not shown). However, if split into ER-positive or ER-negative tumors, *PTEN* gene expression was not significantly higher in neither group in tumors with pathway activation. Akt phosphorylation was significantly more prevalent in tumors harboring *PIK3CA* mutations (27 out of 38 tumors), as compared to *PIK3CA* wild-type tumors (55 out of 132 tumors; *p* = 0.002, data not shown). However, there was no correlation between *PIK3CA* mutation status and the proportion of tumors with phosphorylation of S6K (Ser371), S6K (Thr389), or 4EBP1 further downstream in the PI3K pathway. In TNBC, a high frequency of absent PTEN staining, and low level of Akt-S6K-4EBP1 phosphorylation was observed, as expected for this breast cancer subtype (Online Resource 2). However, there was no significant difference in PTEN staining between TNBC and non-TNBC tumors (data not shown).

### Predictive variables toward chemotherapy response

No association was recorded between pretreatment *PTEN* or *PTENP1* gene expression and response to neither of the chemotherapies given (*n* = 320 patients with stage 3/4 disease), irrespective of *TP53* mutation, *PIK3CA* mutation, HER2 or ER status (data not shown). Furthermore, no association between *PIK3CA* mutation status and response to chemotherapies was detected across the three trials (*n* = 267). Finally, the protein staining intensity for PTEN (*n* = 179), phosphorylated Akt (*n* = 178), S6K (Ser 371, *n* = 173), S6K (Thr 389, *n* = 183), and 4EBP1 (*n* = 175), yielded no predictive information toward chemotherapy response among patients in Study 3.

### Prognostic impact of *PTEN* gene expression

**Fig. 3:**
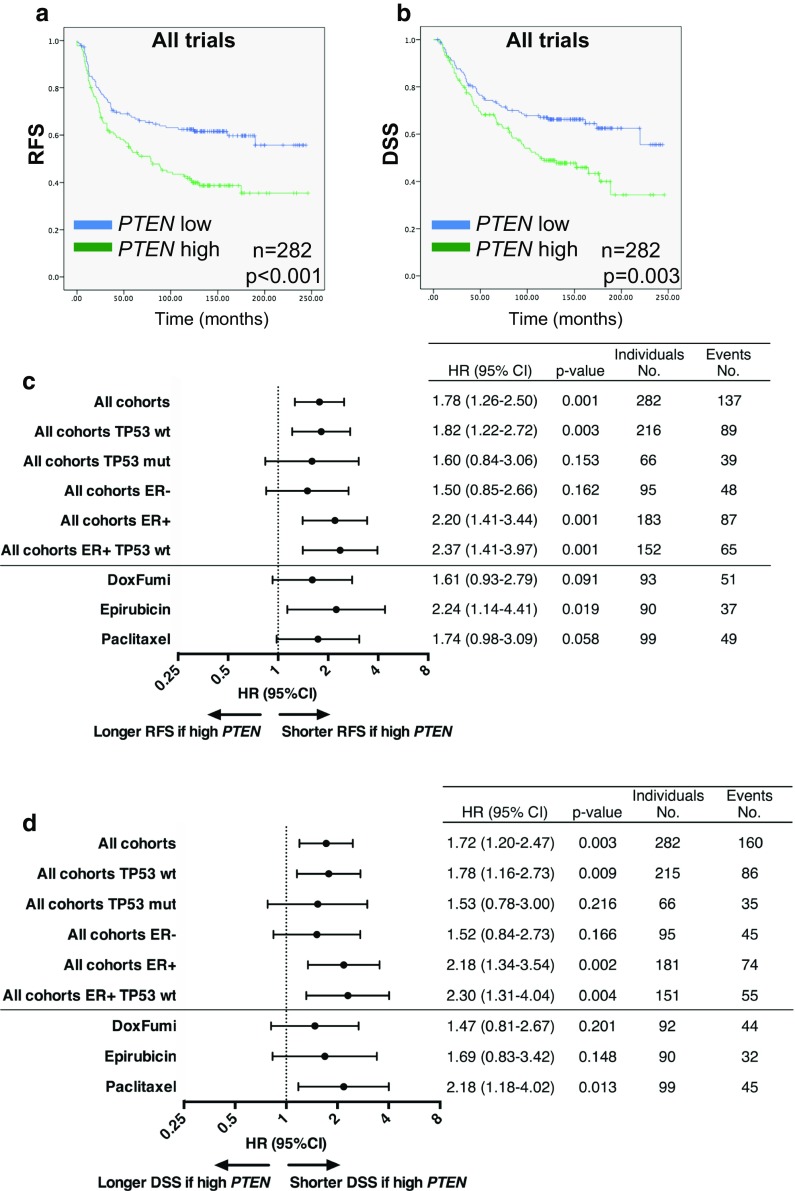
**a**–**b** Recurrence-free (RFS) and disease-specific survival (DSS) after neoadjuvant chemotherapy in patients with locally advanced breast cancer after neoadjuvant epirubicin, paclitaxel, doxorubicin, or 5-FU/mitomycin (FUMI), Studies 1–3 combined. Groups are split by *PTEN* gene expression above or below the median. Censored values are marked with +. n indicates the number of patients used for the survival analysis. **c**–**d** Forest plot for the association between tumor *PTEN* gene expression level and recurrence-free (**c**) or disease-free survival (**d**) in patients with locally advanced breast cancer. Results are presented as individual hazard ratios (HRs) with corresponding 95% confidence intervals (CIs). HR > 1 indicates that the survival of patients with tumor *PTEN* gene expression above the median (*PTEN* high) is shorter than that of patients with *PTEN* low tumors, while HR < 1 indicates the opposite. *RFS* recurrence-free survival, *DSS* disease-specific survival, *wt* wild-type, *mut* mutated, *ER* estrogen receptor

**Fig. 4:**
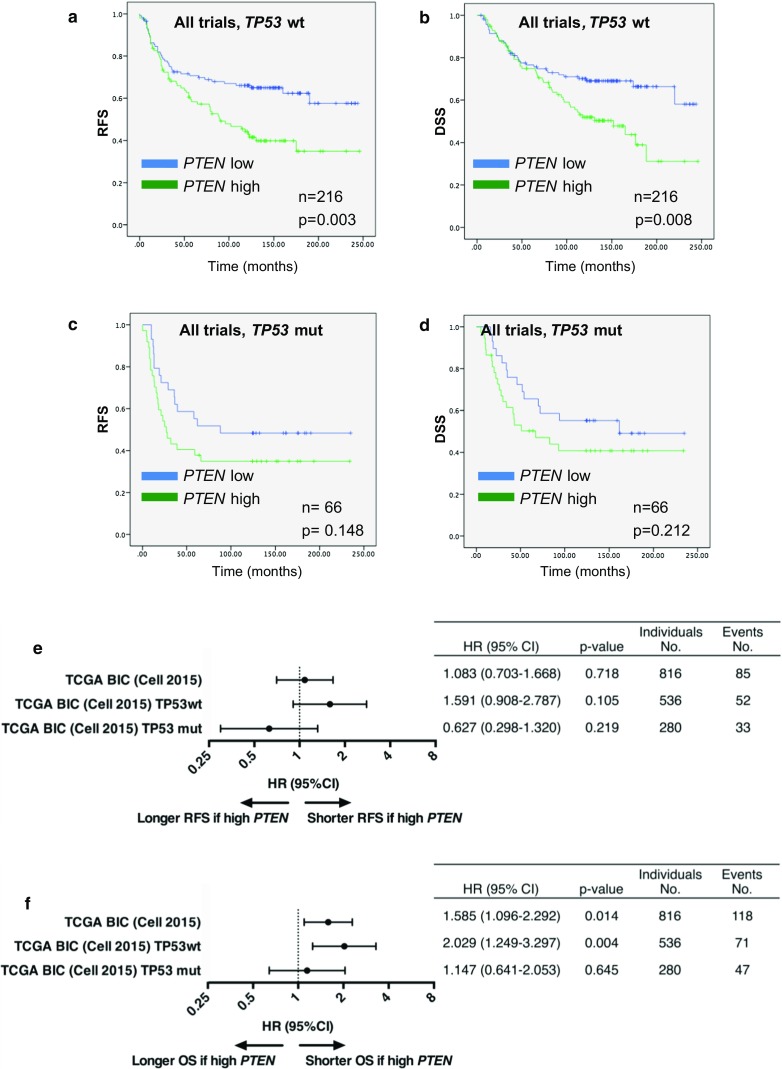
**a**–**d** Recurrence-free (RFS) and disease-specific survival (DSS) after neoadjuvant chemotherapy in patients with locally advanced breast cancer after neoadjuvant epirubicin, paclitaxel, doxorubicin, or 5-FU/mitomycin (FUMI), Studies 1–3 combined. Groups are split by *PTEN* gene expression above or below the median, and stratified by *TP53* mutation status. Censored values are marked with +. *n* indicates the number of patients used for the survival analysis. **e**–**f** Forest plot for the association between tumor *PTEN* gene expression level and recurrence-free (**e**) or overall survival (**f**) in patients with early breast cancer with data extracted from the *The Cancer Genome Atlas (TCGA) Breast Invasive Carcinoma (Cell, 2015)* cohort. Results are presented as individual hazard ratios (HRs) with corresponding 95% confidence intervals (CIs). HR > 1 indicates that the survival of patients with tumor *PTEN* gene expression above the median (*PTEN* high) is shorter than that of patients with *PTEN* low tumors, while HR < 1 indicates the opposite. *RFS* recurrence-free survival, *OS* overall survival, *wt* wild-type, *mut* mutated

Excluding patients with stage 4 disease from the analysis, high *PTEN* gene expression, defined as a *PTEN* mRNA level above the median, was associated with significantly shorter RFS (hazard ratio (HR) for recurrence 1.78, 95% confidence interval (CI) 1.26–2.50, *p* = 0.001), and DSS (HR for breast cancer-specific death 1.72, 95% CI 1.20–2.47, *p* = 0.003) across the pooled cohort of patients with stage 3 disease (*n* = 282, Fig. 3a–d). Among tumors wild-type for *TP53*, a high *PTEN* level remained a negative prognostic marker, with inferior RFS as well as DSS (HR 1.82, 95% CI 1.22–2.72, *p* = 0.003 and HR 1.78, 95% CI 1.16–2.73, *p* = 0.009, respectively; Figs. 3c, d, 4a, b). In contrast, no significant association between outcome and *PTEN* gene expression level was observed in patients with tumors harboring *TP53* mutations (Fig. 3c, d, 4c, d). These findings were consistent across each individual trial (Online Resource 3).

If stratified by ER status, high intratumoral *PTEN* gene expression was associated with inferior RFS (HR 2.20, 95% CI 1.41–3.44, *p* = 0.001) and DSS (HR 2.18, 95% CI 1.34–3.54, *p* = 0.002) among patients with ER-positive tumors only; no effect was observed among patients harboring ER negative tumors (Fig. 3c, d). Moreover, the negative prognostic impact of a high *PTEN* level was evident only in ER-positive tumors harboring wild-type *TP53* (Fig. 3c, d), with inferior RFS (HR 2.37, 95% CI 1.41–3.97, *p* = 0.001) and DSS (HR 2.30, 95% CI 1.31–4.04, *p* = 0.004). No prognostic impact of *PTEN* mRNA level was recorded in patients with ER-negative tumors, irrespective of *TP53* status (Fig. 3c, d). In contrast, *PTEN* gene expression above the median was associated with inferior survival outcome among both HER2 negative (RFS; HR 1.69, 95% CI 1.07–2.69, *p* = 0.026, DSS; HR 1.63, 95% CI 0.99–2.65, *p* = 0.053) and HER2-positive tumors (RFS; HR 2.52, 95% CI 1.07–5.91, *p* = 0.034, DSS; HR 3.16, 95% CI 1.19–8.39, *p* = 0.021, Fig. 3c, d).

Finally, the negative prognostic impact of high *PTEN* mRNA levels was observed exclusively for *PIK3CA* wild-type tumors (RFS; HR 1.89, 95% CI 1.23–2.91, *p* = 0.004, DSS; HR 1.94, 95% CI 1.33–3.07, p = 0.005), with no impact of *PTEN* level in *PIK3CA* mutated tumors (Online Resource 3).

Patients with stage 4 disease (*n* = 44) were excluded from the above survival analysis. However, a high *PTEN* gene expression was associated with significantly shorter DSS (HR for breast cancer-specific death 2.06, 95% CI 1.08–3.01, *p* = 0.027) also for patients with primary metastatic disease (data not shown).

### Validation using the cancer genome atlas (TCGA) public dataset

To validate our findings in another patient cohort, *PTEN* gene expression data were extracted from the cBioPortal database [20, 21], and normalized to *RPLP2* expression in the same dataset. These gene expression data are based on RNA sequencing in the *Breast Invasive Carcinoma (Cell 2015)* analysis [22], which are in whole based upon data generated by the TCGA Research Network: http://cancergenome.nih.gov/. Patient outcome for 816 patients with primary breast cancer was compared for tumors with *PTEN* mRNA levels above or below the median. A negative prognostic impact of high *PTEN* gene expression was observed for overall survival (OS) (HR 1.59, 95% CI 1.10–2.29, *p* = 0.014), but not for RFS (Fig. 4e, f). Among tumors wild-type for *TP53*, a high *PTEN* level remained a negative prognostic marker, with inferior OS (HR 2.03, 95% CI 1.25–3.30, *p* = 0.004; Fig. 4e, f). In contrast, no prognostic value was established for *PTEN* gene expression in tumors harboring *TP53* mutations. DNA sequencing data from the same cohort identified *PTEN* mutations in 42 tumors (5.1%), and 13 tumors thereof exhibited *PTEN* gene expression above and 29 tumors exhibited *PTEN* gene expression below the median. A weak negative correlation (*r*_s_ = −0.090, *p* = 0.010) was established between the presence of *PTEN* mutations and the corresponding *PTEN* gene expression level in the 816 tumors from the TCGA dataset.

### Other prognostic variables

No survival difference was observed between patients with tumor *PTENP1* gene expression above or below the median within the pooled cohort of patients with stage 3 disease, nor within any of the subgroups (Online Resource 4). Also, there was no prognostic impact of *PTENP1* mRNA level in patients with stage 4 disease (data not shown). Similarly, no prognostic impact of either *PIK3CA* mutation status (*n* = 238), PTEN protein expression level (*n* = 168), phosphorylated Akt (*n* = 167), S6K (*n* = 162), or 4EBP1 (*n* = 165) assessed by immunohistochemistry was recorded with respect to RFS and DSS for patients with stage 3 disease (Online Resource 5). Further, in patients with stage 4 disease where tissue was available for IHC (*n* = 18), no correlation was observed between PTEN protein expression and DSS (data not shown).

### Multivariate analysis

Multivariate analysis revealed *PTEN* expression level and *TP53* mutation status to be independent prognostic variables for RFS as well as DSS (Table 2). No significant interaction between *PTEN* mRNA level and *TP53* status with respect to outcome was recorded (Table 2).

**Table 2:** Prognostic indicators of survival by multivariate analysis

Variable	Recurrence-free survival			Disease-specific survival		
	HR (95% CI)	*p* value	Events/patients	HR (95% CI)	*p* value	Events/patients
*PTEN* low	1.00	0.040	57/147	1.00	0.005	51/146^a^
*PTEN* high	1.48 (1.02–2.14)		80/135	1.69 (1.17–2.42)		70/135
*TP53* wt	1.00	0.001	98/216	1.00	0.040	86/215^a^
*TP53* mut	1.75 (1.24–2.46)		39/66	1.51 (1.02–2.24)		35/66
Interaction PTEN*TP53		0.927			0.776	

The parameters included in the multivariate analysis were *PTEN* gene expression (high vs. low) and *TP53* mutation status (wild-type vs. mutated)

*wt* wild-type, *mut* mutated, *HR* hazard ratio, *CI* confidence interval

^a^One case censored before the earliest event in a stratum for disease-free survival

## Discussion

*TP53* inactivating mutations are associated with resistance to anthracycline- and mitomycin-containing chemotherapy and poor prognosis in patients with locally advanced breast cancer [1–7]. Among *TP53* wild-type breast cancers revealing primary resistance to anthracyclines, mutations in the p53 upstream activator *CHEK2* [23] or low expression levels of *ATM* [24] have been observed. Yet, additional factors are known to influence p53 activation in response to genotoxic stress [25, 26]. One such factor is the PTEN protein encoded by the *PTEN* gene [10]. In the present work, we provide data demonstrating the negative prognostic role of high *PTEN* gene expression levels in tumor tissue from patients with locally advanced breast cancer. Notably, the prognostic role of *PTEN* was observed exclusively in patients whose tumors contain preserved *TP53* wild-type status, in accordance with the known functional crosstalk between PTEN and p53 [16, 25, 27–29]. Moreover, our data suggest that the biological impact of *PTEN* in human breast cancer is mediated via mRNA interactions, given a lack of prognostic impact of PTEN protein staining, and a lack of correlation between PTEN and PI3K-Akt-mTOR signaling activity.

To the best of our knowledge, the prognostic role of *PTEN* gene expression by qPCR has not been assessed in patients with breast cancer previously. In a study of 70 patients with stage 2 breast cancer, a gene expression profile of “PTEN loss,” including reduced *PTEN* gene expression, was predictive of poor survival, whereas PTEN protein staining had no prognostic value [30]. However, *PTEN* gene expression was categorized only as up- or downregulated in this microarray analysis, with no further quantification [30]. Another study found *PTEN* gene expression to be significantly higher in 93 human breast cancer samples as compared to healthy breast tissue; however, the potential impact on survival was not assessed [31].

While our clinical data are provocative to suggest a negative prognostic role of high intratumoral *PTEN* gene expression in patients with stage 3 breast cancer, our findings were confirmed by mining the TCGA dataset, to extract RNA sequencing data from 816 patients with stage 1–3 breast cancer [22]. Again, inferior overall survival was observed among patients with high intratumoral *PTEN* mRNA levels, and in particular, for patients with *TP53* wild-type tumors. In this validation cohort, recurrence-free survival did not differ for patients with high versus low *PTEN* levels, as opposed to our findings. This could be attributed to a high proportion of stage 1–2 breast cancer in the TCGA cohort (74%), with a better prognosis, regardless of *PTEN* gene expression, compared to patients with high-risk stage 3 disease in our trials.

The biological reason why high *PTEN* gene expression was associated with an inferior prognosis in our clinical material remains to be elucidated. While a weak correlation between *PTEN* gene expression and PTEN protein staining was observed, PTEN protein levels had no prognostic impact, pointing to biological interactions at the mRNA level as a probable reason.

Firstly, PTEN and p53 influence each other at the transcriptional level as well as through protein interaction [25]. Apart from binding to and stabilizing the p53 protein [16], PTEN inhibits *MDM2* transcription, thus reducing MDM2-mediated p53 degradation [27]. Furthermore, p53 binds to the genomic *PTEN* locus and increases *PTEN* transcription [28, 29]. Notably, while we found *PTEN* and *TP53* to correlate at the mRNA expression level, this was observed among tumors harboring wild-type *TP53* only. Similar, *PTEN* expression correlated to outcome only among *TP53* wild-type tumors. Interestingly, in vitro data indicate that nuclear PTEN modulates the response to genotoxic stress by control of DNA repair in cancer cells with preserved p53 function [32]. While the role of PTEN as a regulator of PI3K cytoplasmic signaling has been extensively studied, the role of nuclear PTEN to influence cell cycle arrest and DNA repair remains less defined [33, 34]. However, the prognostic impact of PTEN protein staining did not differ if nuclear staining was assessed separately, as opposed to combined nuclear and cytoplasmic staining in the current patient cohort.

Secondly, *PTEN* mRNA share miRNA binding sites with multiple gene transcripts implicated in cancer progression [35], and high *PTEN* gene expression could skew the balance between these transcripts in a pro-tumorigenic manner by adsorbing miRNAs which would otherwise target and degrade important oncogenes [36]. Moreover, *PTEN* and the protein non-coding *PTEN pseudogene (PTENP1)* share multiple miRNA binding sites [17], and altering the *PTEN* mRNA level could influence *PTENP1* degradation by competing for the same miRNAs [17, 35]. *PTEN* and *PTENP1* could even interact via *PTENP1* antisense transcripts which bind to the *PTEN* promoter and reduce *PTEN* mRNA expression [37]. While being protein non-coding, *PTENP1* transcripts are biologically active and tumor suppressive in various solid cancers [17, 38–40]. Loss of *PTENP1* on chromosome 9p was identified in 11 out of 118 human breast cancers in data extracted from array-based comparative genomic hybridization databases by Poliseno et al. [17].

To the best of our knowledge, we present the first analysis of *PTENP1* gene expression in human breast cancer. We found *PTENP1* to be expressed in 222 out of 318 human breast cancer samples analyzed. However, the positive correlation between *PTEN* and *PTENP1* transcript levels established in the current report, and the known tumor inhibitory role of *PTENP1*, do not indicate that the negative prognostic impact of high *PTEN* levels is mediated via its pseudogene. Accordingly, no prognostic impact of *PTENP1* was observed in univariate analysis in our patient cohort.

Thirdly, methodological issues associated with immunohistochemistry, such as formalin fixation, antigen retrieval, antibody specificity, and inter-observer variability could explain the lack of strong correlation between *PTEN* mRNA and PTEN protein levels. In comparison, *PTEN* mRNA analysis was performed using a standardized qPCR assay with specific primers and validated PCR products which were quantified independently of the observers.

PTEN is a known inhibitor of the growth-promoting PI3 K-Akt-mTOR pathway [9, 41], and lack of PTEN protein expression is generally associated with increased PI3K-Akt-mTOR signaling [9, 42]. While a significant association between PTEN and phosphorylated Akt by IHC was established previously in 655 breast cancers [43], such an association was not observed in another patient cohort [44], and there was no correlation between the loss of PTEN staining and increased Akt phosphorylation in neither of these two trials [43, 44]. In our current TMA analysis, negative PTEN staining was not associated with increased Akt or S6K phosphorylation levels in 163 locally advanced breast cancers, clearly indicating a lack of biological interaction between PTEN and the PI3K-Akt-mTOR pathway in this setting.

The lack of prognostic impact of PTEN protein expression among 168 patients in the current study is in accordance with several large clinical trials in early breast cancer [30, 43–46]. In the recent CLEOPATRA trial in HER2-positive metastatic breast cancer, a low PTEN protein expression was associated with worse OS, but at the same time an improved progression-free survival, whereas the presence of *PIK3CA* mutations was a definite negative prognostic marker [47]. In the BOLERO-2 trial, patients with ER-positive metastatic breast cancer experienced the same survival benefit from adding the mTOR inhibitor everolimus to exemestane, regardless of “PI3K activation”, defined as low PTEN staining, or *AKT1*, *PIK3CA*, *PIK3R1* or *PTEN* mutations [48]. Finally, the prognostic impact of *PIK3CA* in breast cancer is not well established [49], and our data are consistent with the findings in a recent study, reporting no influence of *PIK3CA* mutation status on survival outcome among 1008 patients with breast cancer at high risk of relapse [50].

## Conclusions

We establish that high *PTEN* gene expression in locally advanced human breast cancers is a marker of poor prognosis, across three neoadjuvant trials with 282 patients. Furthermore, the prognostic impact of *PTEN* gene expression is evident only among patients with *TP53* wild-type breast cancers. This should be examined further to assess whether the outcome of patients with these breast cancer characteristics could be improved by alternative therapeutic measures in the future.

## Electronic supplementary material

Below is the link to the electronic supplementary material.

Supplementary material 1 (PDF 121 kb)

Supplementary material 2 (PDF 124 kb)

Supplementary material 3 (PDF 4461 kb)

Supplementary material 4 (PDF 4508 kb)

Supplementary material 5 (PDF 1882 kb)
