# Supplementary material for: High PTEN gene expression is a negative prognostic marker in human primary breast cancers with preserved p53 function
Source: Breast Cancer Res Treat. 2017 Feb 17;163(1):177–90. doi: 10.1007/s10549-017-4160-5 (PMC5387035; doi:10.1007/s10549-017-4160-5)
Supplement: Supplementary file 1 — Supplementary material 1 (PDF 121 kb) [file 10549_2017_4160_MOESM1_ESM.pdf]

High *PTEN* gene expression is a negative prognostic marker in human primary breast cancers with preserved p53 function

Breast Cancer Research and Treatment

Authors:

Synnøve Yndestad[1,2], Eilin Austreid[1], Stian Knappskog[1,2], Ranjan Chrisanthar[3], Peer Kåre Lilleng[4,5], Per Eystein Lønning[1,2], Hans Petter Eikesdal[1,2*]

Author details:

[1]Section of Oncology, Department of Clinical Science, University of Bergen, Bergen, Norway.

[2]Department of Oncology, Haukeland University Hospital, Bergen, Norway.

[3]Section of Molecular Pathology, Department of Pathology, Oslo University Hospital, Oslo, Norway.

[4]Department of Pathology, Haukeland University Hospital, Bergen, Norway.

[5]Laboratory of Pathology, Department of Clinical Medicine, University of Bergen, Bergen, Norway.

***Correspondence:** hans.eikesdal@k2.uib.no

Online Resource 1.

Basic genomic procedures

RNA was extracted from breast cancer samples using either Trizol (Invitrogen) (Studies 1-2) or the Qiagen RNeasy protocol (Study 3). RNA concentrations were measured by a NanoDrop spectrophotometer (Thermo Scientific), and cDNA was made from 500 ng of RNA using Transcriptor Reverse Transcriptase kit w/random hexamers (Roche). DNA for *PTEN* and *PIK3CA* analysis was extracted using the Qiagen DNeasy kit.

Real-time qPCR was performed using dual labeled hydrolysis probes (TIB MOLBIOL). The Light Cycler 480 (Roche) was used to detect the PCR products. Lack of gene expression was defined as lack of gene amplification after 37 cycles. Gene of interest was given as a ratio to the housekeeping gene *RPLP2*, and as a mean of three independent runs, normalized to a cDNA pool of 6 breast cancer cell lines. *PTEN* was amplified much earlier than *PTENP1* during the qPCR procedure, demonstrating higher absolute levels of *PTEN* than *PTENP1* transcripts. The relative *PTENP1/RPLP2* gene expression was therefore divided by 98 ($2^{\triangle Cp}$) since *PTENP1* appeared on average 6.62 cycles after *PTEN* (ΔCp) on real-time RT-PCR.

For each of the three trials patients were sorted based on increasing *PTEN* or *PTENP1* gene expression in the tumors, and the groups were split by gene expression above or below the median.

Gene expression of *PTEN* and the known transcribed, processed *PTENP1* [1] was analyzed using cDNA produced from DNAse-treated RNA. Due to the 98% sequence homology between *PTEN* and *PTENP1* [1], the specificity of all PCR reactions was verified by Sanger sequencing.

PTEN 3´UTR primers from [2]:

PTEN_F2: CTTCTCCATCTCCTGTGTAATCAA

PTEN_R2: GTTGACTGATGTAGGTACTAACAGCAT

PTEN_FAM: FAM6-CCAGTGCTAAAATTCA-BBQ

PTENP1 3´UTR:

PTENP1_F18: TGCAGTTAGCTAAGAGAAGTTTCTG

PTENP1_R20: CCATTCCCCTAACCCAAATAC

PTENP1_FAM: FAM6-AGGGTTTTGCTGCATTCTTGCAT-BBQ

RPLP2:

RPLP2_F GACCGGCTCAACAAGGTTAT

RPLP2_R CCCCACCAGCAGGTACAC

RPLP2_Cy5 Cy5-AGCTGAATGGAAAAAACATTGAAGACGTC-BBQ

TP53:

TP53_F CGAGCACTGCCCAACAA

TP53_A GCCTCATTCAGCTCTCGGAA

TP53_TM FAM6-CACGGATCTGAAGGGTGAAATATTCTCCA—BBQ

Hot-spot *PIK3CA* mutations in exons 10 and 21 (previously exon 9 and 20) [3] were assessed in 275 pre-treatment breast cancer samples from Studies 1-3; tumor DNA was used as PCR template with the primers listed below. *PTEN* mutation status was assessed in 183 pre-treatment breast cancer samples from Study 3, using cDNA as the template for nested PCR with the primers listed below. PCR products were analyzed at Center for Medical Genetics and Molecular Medicine, Haukeland University Hospital, by Sanger sequencing using the BigDye v1.1 reaction mix (Applied Biosystems).

PIK3CA sequencing primers:

PI3KCA_ex10_FTGAAAATGTATTTGCTTTTTCTG

PI3KCA_ex10_R ACATGCTGAGATCAGCCAAA

PI3KCA_ex10_F2 GGGAAAAATATGACAAAGAAAGC

PI3KCA_ex10_R2 CTGAGATCAGCCAAATTCAGTT

PI3KCA_ex10_F3 GGAAAAATATGACAAAGAAAGCTATATAAG

PI3KCA_ex10_R3 ACAGAGAATCTCCATTTTAGCAC

PI3KCA_ex21_FCATTTGCTCCAAACTGACCA

PI3KCA_ex21_R CCTGCTGAGAGTTATTAACAGTGC

PI3KCA_ex21_F2 GCTCCAAACTGACCAAACTGTTC

PI3KCA_ex21_R2 TGGAATCCAGAGTGAGCTTTC

PI3KCA_ex21_seqF	CTCAATGATGCTTGGCTCTG
PI3KCA_ex21_seqR	AGAAAATGAAAGCTCACTCTG
PI3KCA_ex21_seqF0	GGAGATGTGTTACAAGGCTTATCTA
PI3KCA_ex21_seqR2	GCATTGAACTGAAAAGATAACTGAGAAA

PTEN sequencing primers (nested PCR):

PTEN outerF	TCCAGAGCCATTTCCATC
PTEN outerR	TGTCAAAACCCTGTGGATG
PTEN inner primerF	CTCCTCCTTTTTCTTCAGC
PTEN inner primerR	CAAGAGGGATAAAACACCAT
PTEN sequencingF	GAGTAACTATTCCCAGTCAGAGG
PTEN sequencingR	AACTGAGGATTGCAAGTTCC

Immunohistochemistry (IHC) and *in situ* hybridization (ISH)

Tissue microarrays (TMAs) were created from Study 3 with quadruplicate 1 mm cores from formalin-fixed paraffin embedded (FFPE) tumor material collected at the time of diagnosis. Briefly, tumor sections (4 μm) from the TMAs were de-paraffinized and rehydrated, before antigen retrieval at 98°C for 1 hour in DAKO Target Retrieval Buffer (pH 6.0 or 9.0). After blocking with diluted goat serum for 30 min, the slides were incubated overnight (+4°C) with the primary antibody. After blocking endogenous peroxidase activity for 15 min with DAKO peroxidase block, a biotinylated goat anti-rabbit secondary antibody was applied for 30 min (Vector Laboratories). The antigen-antibody complex was revealed with avidin-biotin-peroxidase (ABC) for 30 min according to the manufacturer's instructions (Vectastain® ABC Kit, Vector). The immune reaction was visualized by incubation with diamino-benzidine tetrahydrochloride (Vector). The sections were then counterstained with haematoxylin (Fisher), dehydrated and mounted with Entellan (Electron Microscopy Services). Parallel sections were run for all the experiments without primary antibody, to assure the specificity of the immune reactions.

HER2 assessment was performed according to international guidelines [4], using the Ventana-Roche HercepTest and the INFORM HER2 Dual ISH DNA Probe Cocktail (Roche) assays. Briefly, HER2 immunostaining (HercepTest) scores 0 and 1 were classified as HER2 negative, score 2 as equivocal and score 3 as HER2 positive. Biopsies with equivocal results were analyzed by dual-color dual-hapten brightfield *in situ* hybridization (DDISH, Ventana-Roche) using the INFORMER HER2 Dual ISH DNA Probe Cocktail, the UltraView Red ISH DIG Detection and ultraView SISH DNP Detection Kits. HER2 and chromosome 17 centromere (CEP17) were counted in 20 tumor cell nuclei and specimens with equivocal ratio HER2/CEP17 (1,8-2,2) an additional 20 nuclei were counted. A ratio below 1.8 was considered negative for Her2, and above 1.8 was considered HER2 amplified and therefore HER2 positive.

References:

1. Dahia PL, FitzGerald MG, Zhang X, Marsh DJ, Zheng Z, Pietsch T, von Deimling A, Haluska FG, Haber DA, Eng C (1998) A highly conserved processed PTEN pseudogene is located on chromosome band 9p21. Oncogene 16 (18):2403-2406. doi:10.1038/sj.onc.1201762

2. Poliseno L, Salmena L, Zhang J, Carver B, Haveman WJ, Pandolfi PP (2010) A coding-independent function of gene and pseudogene mRNAs regulates tumour biology. Nature 465 (7301):1033-1038. doi:10.1038/nature09144

3. Samuels Y, Wang Z, Bardelli A, Silliman N, Ptak J, Szabo S, Yan H, Gazdar A, Powell SM, Riggins GJ, Willson JK, Markowitz S, Kinzler KW, Vogelstein B, Velculescu VE (2004) High frequency of mutations of the PIK3CA gene in human cancers. Science 304 (5670):554. doi:10.1126/science.1096502

4. Wolff AC, Hammond ME, Hicks DG, Dowsett M, McShane LM, Allison KH, Allred DC, Bartlett JM, Bilous M, Fitzgibbons P, Hanna W, Jenkins RB, Mangu PB, Paik S, Perez EA, Press MF, Spears PA, Vance GH, Viale G, Hayes DF, American Society of Clinical O, College of American P (2013) Recommendations for human epidermal growth factor receptor 2 testing in breast cancer: American Society of Clinical Oncology/College of American Pathologists clinical practice guideline update. J Clin Oncol 31 (31):3997-4013. doi:10.1200/JCO.2013.50.9984
