# Supplementary material for: High PTEN gene expression is a negative prognostic marker in human primary breast cancers with preserved p53 function
Source: Breast Cancer Res Treat. 2017 Feb 17;163(1):177–90. doi: 10.1007/s10549-017-4160-5 (PMC5387035; doi:10.1007/s10549-017-4160-5)
Supplement: Supplementary file 2 — Supplementary material 2 (PDF 124 kb) [file 10549_2017_4160_MOESM2_ESM.pdf]

High *PTEN* gene expression is a negative prognostic marker in human primary breast cancers with preserved p53 function

Breast Cancer Research and Treatment

Authors:

Synnøve Yndestad[1,2], Eilin Austreid[1], Stian Knappskog[1,2], Ranjan Chrisanthar[3], Peer Kåre Lilleng[4,5], Per Eystein Lønning[1,2], Hans Petter Eikesdal[1,2*]

Author details:

[1]Section of Oncology, Department of Clinical Science, University of Bergen, Bergen, Norway.

[2]Department of Oncology, Haukeland University Hospital, Bergen, Norway.

[3]Section of Molecular Pathology, Department of Pathology, Oslo University Hospital, Oslo, Norway.

[4]Department of Pathology, Haukeland University Hospital, Bergen, Norway.

[5]Laboratory of Pathology, Department of Clinical Medicine, University of Bergen, Bergen, Norway.

***Correspondence:** hans.eikesdal@k2.uib.no

Online Resource 2.

		All samples		ER+		ER-		TNBC		TP53 wt		TP53 mut		PIK3CA wt		PIK3CA mut	
	Score	n	%	n	%	n	%	n	%	n	%	n	%	n	%	n	%
PTEN (c+n)*	0	55	29	23	22	32	40	13	36	31	23	18	49	43	32	6	17
	0.5	20	11	9	9	11	14	7	19	15	11	4	11	15	11	4	11
	1	38	20	19	18	18	22	7	19	30	22	6	16	30	22	6	17
	1.5	48	26	33	32	14	17	6	17	40	30	5	14	31	23	14	39
	2	17	9	13	13	4	5	1	3	13	10	3	8	11	8	5	14
	2.5	5	3	4	4	1	1	1	3	4	3	0	0	4	3	0	0
	3	4	2	3	3	1	1	1	3	1	1	1	3	1	1	1	3
	Total	187	100	104	100	81	100	36	100	134	100	37	100	135	100	36	100
PTEN (c)	0	64	34	29	28	35	43	14	39	40	30	18	49	50	37	8	22
	1	88	47	52	50	34	42	17	47	72	54	10	27	63	47	19	53
	2	30	16	20	19	10	12	4	11	21	16	7	19	21	16	7	19
	3	5	3	3	3	2	2	1	3	1	1	2	5	1	1	2	6
	Total	187	100	104	100	81	100	36	100	134	100	37	100	135	100	36	100
PTEN (n)	0	76	41	30	29	46	57	21	58	44	33	25	68	58	43	11	31
	1	40	21	22	21	17	21	6	17	31	23	7	19	30	22	8	22
	2	59	32	43	41	15	19	7	19	52	39	3	8	41	30	14	39
	3	12	6	9	9	3	4	2	6	7	5	2	5	6	4	3	8
	Total	187	100	104	100	81	100	36	100	134	100	37	100	135	100	36	100
pAkt (Ser473) (n)	0	96	52	49	47	46	58	21	60	67	50	21	57	77	58	11	29
	1	50	27	30	29	19	24	10	29	36	27	10	27	34	26	12	32
	2	36	19	23	22	13	16	4	11	28	21	5	14	20	15	13	34
	3	4	2	2	2	2	3	0	0	2	2	1	3	1	1	2	5
	Total	186	100	104	100	80	100	35	100	133	100	37	100	132	100	38	100
pS6K (Ser371) (n)	0	49	27	12	12	36	44	18	50	30	23	16	43	35	27	11	32
	1	53	29	25	26	27	33	12	33	40	31	9	24	41	31	8	24
	2	76	42	58	59	18	22	6	17	56	44	12	32	54	41	14	41
	3	3	2	3	3	0	0	0	0	2	2	0	0	1	1	1	3
	Total	181	100	98	100	81	100	36	100	128	100	37	100	131	100	34	100
pS6K (Thr389) (n)	0	177	93	94	88	81	99	35	97	128	93	34	92	125	93	37	95
	1	9	5	9	8	0	0	0	0	5	4	3	8	6	4	2	5
	2	5	3	4	4	1	1	1	3	4	3	0	0	4	3	0	0
	3	0	0	0	0	0	0	0	0	0	0	0	0	0	0	0	0
	Total	191	100	107	100	82	100	36	100	137	100	37	100	135	100	39	100
p4EBP1 (Thr70) (c)	0	14	8	6	6	8	10	5	14	11	8	2	5	10	8	3	8
	1	70	38	44	44	24	30	8	22	52	40	10	27	44	34	18	49
	2	83	45	41	41	42	52	21	58	56	43	22	59	63	48	15	41
	3	16	9	9	9	7	9	2	6	11	8	3	8	13	10	1	3
	Total	183	100	100	100	81	100	36	100	130	100	37	100	130	100	37	100

Immunohistochemistry results for PTEN and phosphorylated Akt (pAkt, Ser473), S6K (pS6K, Ser371 or Thr389) and 4EBP1 (p4EBP1, Thr70) proteins. Staining was performed on tissue microarray sections of pretreatment breast cancer samples from Study 3, and results are summarized for all samples, or subgrouped based on estrogen receptor (ER) status, triple negative breast cancer status (TNBC;

ER/PGR/HER2 negative breast cancer), *TP53* or *PIK3CA* mutation status. (c) = cytoplasmic staining. (n) = nuclear staining. *PTEN (c+n) score is given as (c+n)/2.
