# Supplementary material for: High PTEN gene expression is a negative prognostic marker in human primary breast cancers with preserved p53 function
Source: Breast Cancer Res Treat. 2017 Feb 17;163(1):177–90. doi: 10.1007/s10549-017-4160-5 (PMC5387035; doi:10.1007/s10549-017-4160-5)
Supplement: Supplementary file 3 — Supplementary material 3 (PDF 4461 kb) [file 10549_2017_4160_MOESM3_ESM.pdf]

High *PTEN* gene expression is a negative prognostic marker in human primary breast cancers with preserved p53 function

Breast Cancer Research and Treatment

Authors:

Synnøve Yndestad[1,2], Eilin Austreid[1], Stian Knappskog[1,2], Ranjan Chrisanthar[3], Peer Kåre Lilleng[4,5], Per Eystein Lønning[1,2], Hans Petter Eikesdal[1,2*]

Author details:

[1]Section of Oncology, Department of Clinical Science, University of Bergen, Bergen, Norway.

[2]Department of Oncology, Haukeland University Hospital, Bergen, Norway.

[3]Section of Molecular Pathology, Department of Pathology, Oslo University Hospital, Oslo, Norway.

[4]Department of Pathology, Haukeland University Hospital, Bergen, Norway.

[5]Laboratory of Pathology, Department of Clinical Medicine, University of Bergen, Bergen, Norway.

***Correspondence:** hans.eikesdal@k2.uib.no

Online Resource 3

a-b Forest plot for the association between tumor *PTEN* gene expression level and recurrence-free (**a**) or disease-free survival (**b**) in patients with locally advanced breast cancer. Results are presented as individual hazard ratios (HRs) with corresponding 95% confidence intervals (CIs) for Study 1 (doxorubicin trial), Study 2 (FUMI trial) and Study 3 (epirubicin/paclitaxel trial) combined (i.e. all cohorts) or split by subgroups. HR>1 indicates that the survival of patients with tumor *PTEN* gene expression above the median (*PTEN* high) is shorter than that of patients with *PTEN* low tumors, while HR<1 indicates the opposite. RFS: recurrence-free survival, DSS: disease-specific survival, wt: wildtype, mut: mutated, ER: estrogen receptor, PGR: progesterone receptor, TNBC: triple negative breast cancer (ER/PGR/HER2 negative breast cancer), *for patients in Study 1 and 2 PGR status was not available, and TNBC was defined as ER/HER2 negative tumors

a

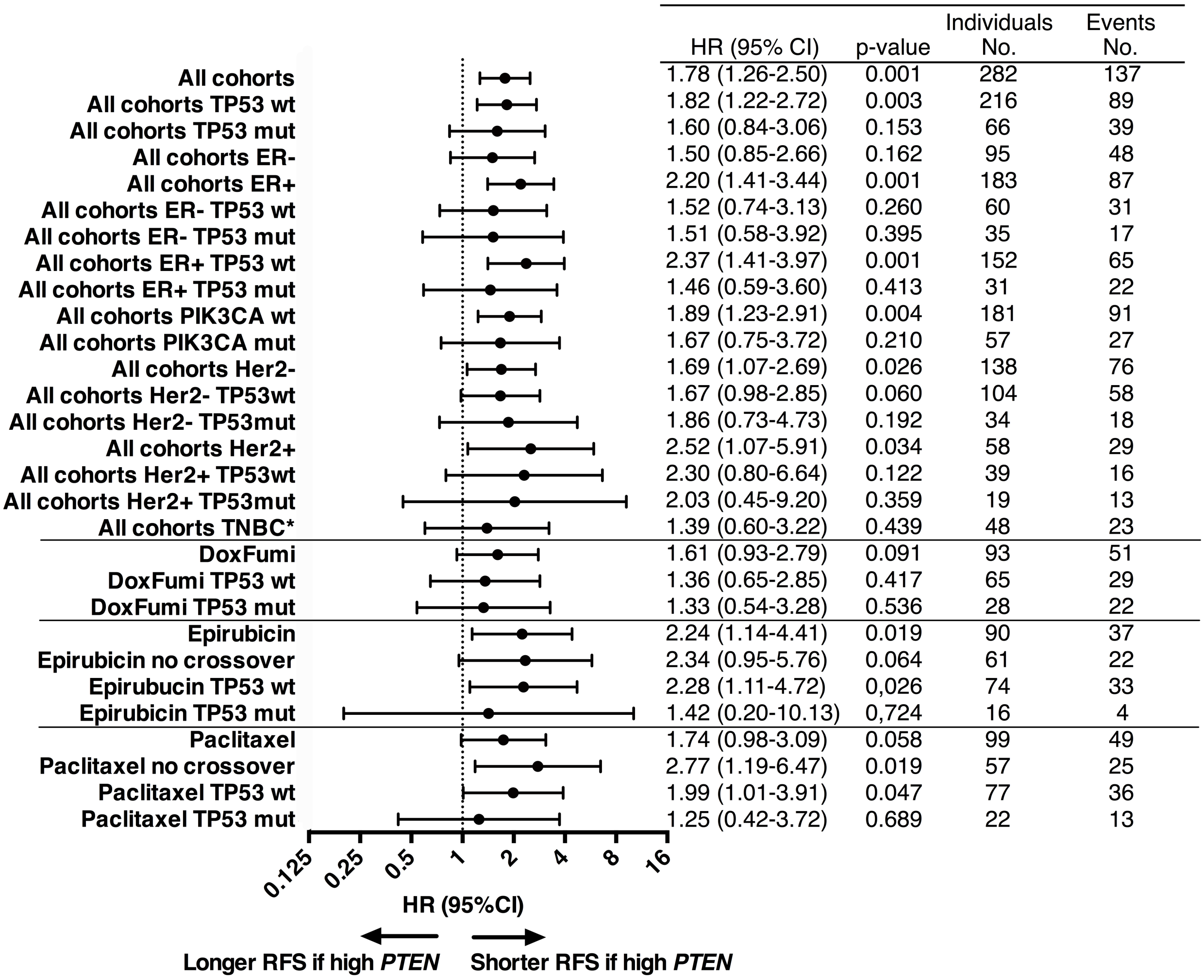

	HR (95% CI)	p-value	Individuals No.	Events No.
All cohorts	1.78 (1.26-2.50)	0.001	282	137
All cohorts TP53 wt	1.82 (1.22-2.72)	0.003	216	89
All cohorts TP53 mut	1.60 (0.84-3.06)	0.153	66	39
All cohorts ER-	1.50 (0.85-2.66)	0.162	95	48
All cohorts ER+	2.20 (1.41-3.44)	0.001	183	87
All cohorts ER- TP53 wt	1.52 (0.74-3.13)	0.260	60	31
All cohorts ER- TP53 mut	1.51 (0.58-3.92)	0.395	35	17
All cohorts ER+ TP53 wt	2.37 (1.41-3.97)	0.001	152	65
All cohorts ER+ TP53 mut	1.46 (0.59-3.60)	0.413	31	22
All cohorts PIK3CA wt	1.89 (1.23-2.91)	0.004	181	91
All cohorts PIK3CA mut	1.67 (0.75-3.72)	0.210	57	27
All cohorts Her2-	1.69 (1.07-2.69)	0.026	138	76
All cohorts Her2- TP53wt	1.67 (0.98-2.85)	0.060	104	58
All cohorts Her2- TP53mut	1.86 (0.73-4.73)	0.192	34	18
All cohorts Her2+	2.52 (1.07-5.91)	0.034	58	29
All cohorts Her2+ TP53wt	2.30 (0.80-6.64)	0.122	39	16
All cohorts Her2+ TP53mut	2.03 (0.45-9.20)	0.359	19	13
All cohorts TNBC*	1.39 (0.60-3.22)	0.439	48	23
DoxFumi	1.61 (0.93-2.79)	0.091	93	51
DoxFumi TP53 wt	1.36 (0.65-2.85)	0.417	65	29
DoxFumi TP53 mut	1.33 (0.54-3.28)	0.536	28	22
Epirubicin	2.24 (1.14-4.41)	0.019	90	37
Epirubicin no crossover	2.34 (0.95-5.76)	0.064	61	22
Epirubucin TP53 wt	2.28 (1.11-4.72)	0,026	74	33
Epirubicin TP53 mut	1.42 (0.20-10.13)	0,724	16	4
Paclitaxel	1.74 (0.98-3.09)	0.058	99	49
Paclitaxel no crossover	2.77 (1.19-6.47)	0.019	57	25
Paclitaxel TP53 wt	1.99 (1.01-3.91)	0.047	77	36
Paclitaxel TP53 mut	1.25 (0.42-3.72)	0.689	22	13

b

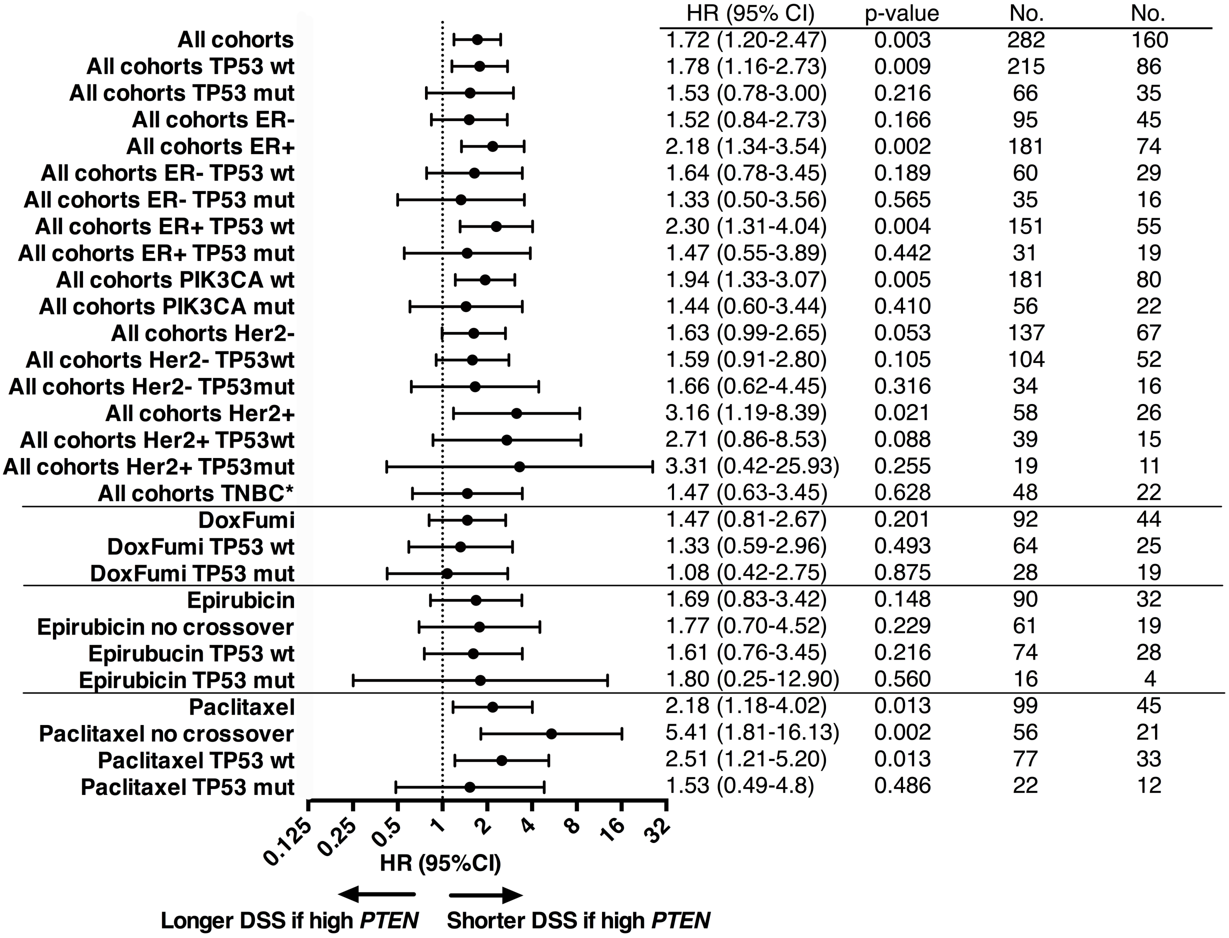

	HR (95% CI)	p-value	Individuals No.	Events No.
All cohorts	1.72 (1.20-2.47)	0.003	282	160
All cohorts TP53 wt	1.78 (1.16-2.73)	0.009	215	86
All cohorts TP53 mut	1.53 (0.78-3.00)	0.216	66	35
All cohorts ER-	1.52 (0.84-2.73)	0.166	95	45
All cohorts ER+	2.18 (1.34-3.54)	0.002	181	74
All cohorts ER- TP53 wt	1.64 (0.78-3.45)	0.189	60	29
All cohorts ER- TP53 mut	1.33 (0.50-3.56)	0.565	35	16
All cohorts ER+ TP53 wt	2.30 (1.31-4.04)	0.004	151	55
All cohorts ER+ TP53 mut	1.47 (0.55-3.89)	0.442	31	19
All cohorts PIK3CA wt	1.94 (1.33-3.07)	0.005	181	80
All cohorts PIK3CA mut	1.44 (0.60-3.44)	0.410	56	22
All cohorts Her2-	1.63 (0.99-2.65)	0.053	137	67
All cohorts Her2- TP53wt	1.59 (0.91-2.80)	0.105	104	52
All cohorts Her2- TP53mut	1.66 (0.62-4.45)	0.316	34	16
All cohorts Her2+	3.16 (1.19-8.39)	0.021	58	26
All cohorts Her2+ TP53wt	2.71 (0.86-8.53)	0.088	39	15
All cohorts Her2+ TP53mut	3.31 (0.42-25.93)	0.255	19	11
All cohorts TNBC*	1.47 (0.63-3.45)	0.628	48	22
DoxFumi	1.47 (0.81-2.67)	0.201	92	44
DoxFumi TP53 wt	1.33 (0.59-2.96)	0.493	64	25
DoxFumi TP53 mut	1.08 (0.42-2.75)	0.875	28	19
Epirubicin	1.69 (0.83-3.42)	0.148	90	32
Epirubicin no crossover	1.77 (0.70-4.52)	0.229	61	19
Epirubucin TP53 wt	1.61 (0.76-3.45)	0.216	74	28
Epirubicin TP53 mut	1.80 (0.25-12.90)	0.560	16	4
Paclitaxel	2.18 (1.18-4.02)	0.013	99	45
Paclitaxel no crossover	5.41 (1.81-16.13)	0.002	56	21
Paclitaxel TP53 wt	2.51 (1.21-5.20)	0.013	77	33
Paclitaxel TP53 mut	1.53 (0.49-4.8)	0.486	22	12
