# Supplementary material for: High PTEN gene expression is a negative prognostic marker in human primary breast cancers with preserved p53 function
Source: Breast Cancer Res Treat. 2017 Feb 17;163(1):177–90. doi: 10.1007/s10549-017-4160-5 (PMC5387035; doi:10.1007/s10549-017-4160-5)

**High *PTEN* gene expression is a negative prognostic marker in human primary breast cancers with preserved p53 function**

**Breast Cancer Research and Treatment**

**Authors:**

Synnøve Yndestad[1,2], Eilin Austreid[1], Stian Knappskog[1,2], Ranjan Chrisanthar[3], Peer Kåre Lilleng[4,5], Per Eystein Lønning[1,2], Hans Petter Eikesdal[1,2*]

**Author details:**

[1]Section of Oncology, Department of Clinical Science, University of Bergen, Bergen, Norway.

[2]Department of Oncology, Haukeland University Hospital, Bergen, Norway.

[3]Section of Molecular Pathology, Department of Pathology, Oslo University Hospital, Oslo, Norway.

[4]Department of Pathology, Haukeland University Hospital, Bergen, Norway.

[5]Laboratory of Pathology, Department of Clinical Medicine, University of Bergen, Bergen, Norway.

***Correspondence:** hans.eikesdal@k2.uib.no

## Online Resource 4

**a-b** Forest plot for the association between tumor *PTEN pseudogene* (*PTENP1)* gene expression level and recurrence-free (**a**) or disease-free survival (**b**) in patients with locally advanced breast cancer. Results are presented as individual hazard ratios (HRs) with corresponding 95% confidence intervals (CIs) for Study 1 (doxorubicin trial), Study 2 (FUMI trial) and Study 3 (epirubicin/paclitaxel trial) combined (i.e. all cohorts) or split by subgroups. HR>1 indicates that the survival of patients with tumor *PTENP1* gene expression above the median (*PTENP1* high) is shorter than that of patients with *PTENP1* low tumors, while HR<1 indicates the opposite. RFS: recurrence-free survival, DSS: disease-specific survival, wt: wildtype, mut: mutated, ER: estrogen receptor, PGR: progesterone receptor, TNBC: triple negative breast cancer (ER/PGR/HER2 negative breast cancer), *for patients in Study 1 and 2 PGR status was not available, and TNBC was defined as ER/HER2 negative tumors. **one case was censored before the earliest event in a stratum

**a**

| | HR (95% CI) | p-value | Individuals No. | Events No. |
|---|---|---|---|---|
| All cohorts | 1.12 (0.80-1.57) | 0.496 | 282 | 137 |
| All cohorts TP53 wt | 1.12 (0.75-1.66) | 0.583 | 216 | 98 |
| All cohorts TP53 mut | 1.08 (0.58-2.02) | 0.811 | 66 | 39 |
| All cohorts ER- | 0.80 (0.45-1.42) | 0.455 | 95 | 48 |
| All cohorts ER+ | 1.43 (0.94-2.18) | 0.098 | 183 | 87 |
| All cohorts ER- TP53 wt | 1.01 (0.50-2.04) | 0.986 | 60 | 31 |
| All cohorts ER- TP53 mut | 0.50 (0.19-1.36) | 0.176 | 35 | 17 |
| All cohorts ER+ TP53 wt | 1.23 (0.75-2.00) | 0.411 | 152 | 65 |
| All cohorts ER+ TP53 mut | 2.22 (0.92-5.33) | 0.076 | 31 | 22 |
| All cohorts PIK3CA wt | 1.14 (0.75-1.72) | 0.539 | 181 | 91 |
| All cohorts PIK3CA mut | 1.09 (0.51-2.33) | 0.816 | 57 | 27 |
| All cohorts Her2- | 0.97 (0.62-1.52) | 0.898 | 138 | 76 |
| All cohorts Her2- TP53wt | 0.84 (0.50-1.41) | 0.516 | 104 | 58 |
| All cohorts Her2- TP53mut | 1.40 (0.56-3.54) | 0.472 | 34 | 18 |
| All cohorts Her2+ | 1.93 (0.91-4.10) | 0.086 | 58 | 29 |
| All cohorts Her2+ TP53wt | 2.45 (0.89-6.75) | 0.083 | 39 | 16 |
| All cohorts Her2+ TP53mut | 1.08 (0.35-3.29) | 0.899 | 19 | 13 |
| All cohorts TNBC* | 0.63 (0.28-1.45) | 0.280 | 48 | 23 |
| DoxFumi | 1.17 (0.67-2.03) | 0.583 | 93 | 51 |
| DoxFumi TP53 wt | 1.14 (0.55-2.37) | 0.721 | 65 | 29 |
| DoxFumi TP53 mut | 1.37 (0.59-3.19) | 0.462 | 28 | 22 |
| Epirubicin | 1.38 (0.72-2.63) | 0.333 | 90 | 37 |
| Epirubicin no crossover | 1.43 (0.61-3.34) | 0.413 | 61 | 22 |
| Epirubucin TP53 wt | 1.22 (0.62-2.43) | 0.558 | 74 | 33 |
| Epirubicin TP53 mut | 3.23 (0.34-31.01) | 0.310 | 16 | 4 |
| Paclitaxel | 0.95 (0.54-1.66) | 0.848 | 99 | 49 |
| Paclitaxel no crossover | 0.98 (0.44-2.19) | 0.958 | 57 | 25 |
| Paclitaxel TP53 wt | 1.01 (0.53-1.95) | 0.972 | 77 | 36 |
| Paclitaxel TP53 mut | 0.66 (0.22-1.97) | 0.452 | 22 | 12 |

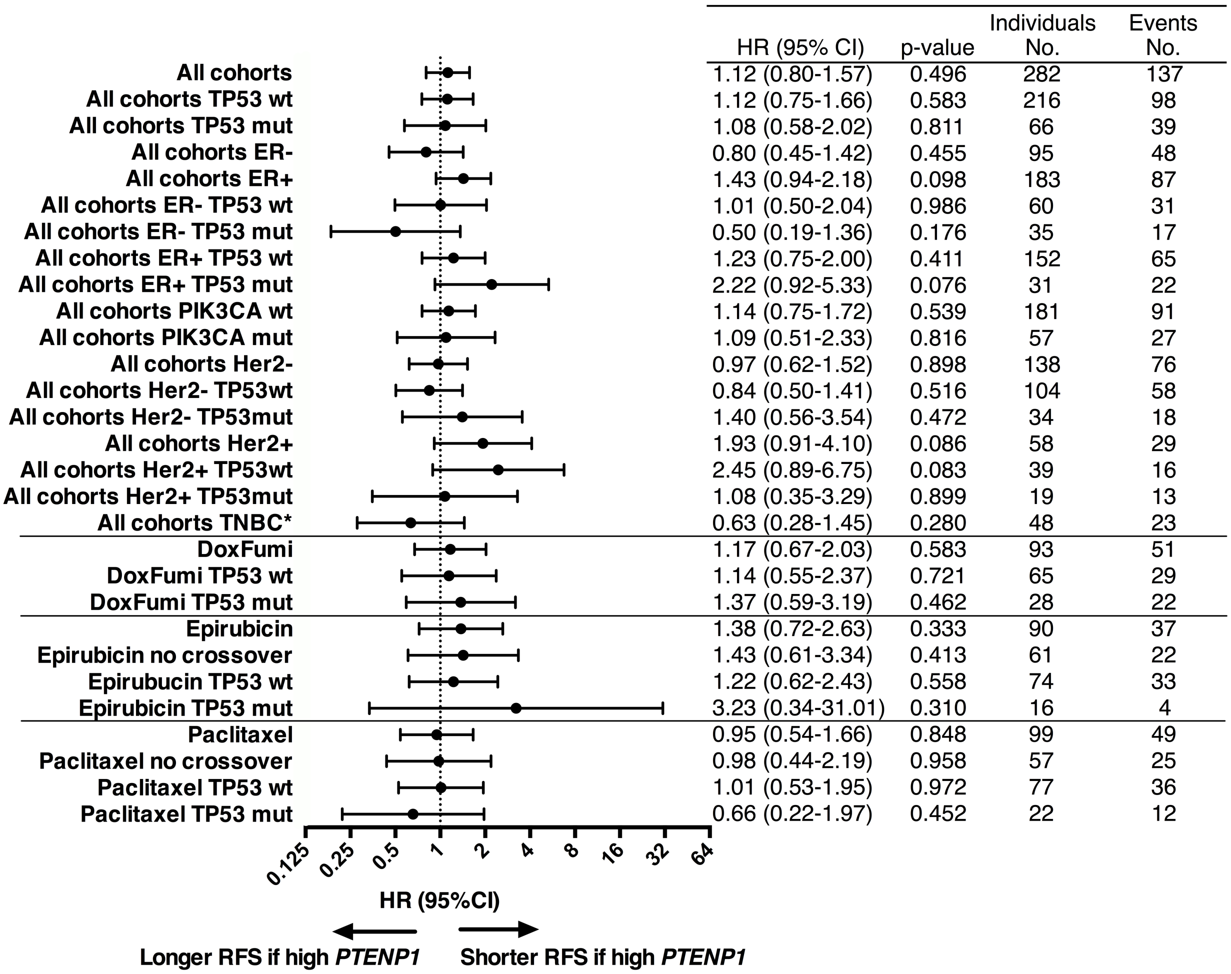


**b**

| | HR (95% CI) | p-value | Individuals No. | Events No. |
|---|---|---|---|---|
| All cohorts | 1.03 (0.72-1.47) | 0.87 | 281** | 121 |
| All cohorts TP53 wt | 1.03 (0.67-1.57) | 0.895 | 215 | 86 |
| All cohorts TP53 mut | 0.98 (0.01-1.91) | 0.982 | 66 | 35 |
| All cohorts ER- | 0.83 (0.46-1.49) | 0.533 | 95 | 45 |
| All cohorts ER+ | 1.24 (0.79-1.96) | 0.355 | 182 | 74 |
| All cohorts ER- TP53 wt | 1.02 (0.49-2.12) | 0.96 | 60 | 29 |
| All cohorts ER- TP53 mut | 0.54 (0.20-1.50) | 0.24 | 35 | 16 |
| All cohorts ER+ TP53 wt | 1.09 (0.64-1.85) | 0.748 | 151 | 55 |
| All cohorts ER+ TP53 mut | 1.72 (0.68-4.35) | 0.256 | 31 | 19 |
| All cohorts PIK3CA wt | 1.04 (0.67-1.61) | 0.875 | 181 | 80 |
| All cohorts PIK3CA mut | 1.11 (0.48-2.56) | 0.816 | 56 | 22 |
| All cohorts Her2- | 0.88 (0.54-1.42) | 0.602 | 137 | 67 |
| All cohorts Her2- TP53wt | 0.75 (0.43-1.30) | 0.310 | 104 | 52 |
| All cohorts Her2- TP53mut | 1.24 (0.46-3.34) | 0.669 | 34 | 16 |
| All cohorts Her2+ | 2.16 (0.96-4.85) | 0.63 | 58 | 26 |
| All cohorts Her2+ TP53wt | 2.78 (0.95-8.15) | 0.062 | 39 | 15 |
| All cohorts Her2+ TP53mut | 1.16 (0.34-3.98) | 0.815 | 19 | 11 |
| All cohorts TNBC* | 0.56 (0.24-1.30) | 0.175 | 48 | 22 |
| DoxFumi | 1.22 (0.67-2.20) | 0.519 | 92 | 44 |
| DoxFumi TP53 wt | 1.30 (0.59-2.85) | 0.514 | 65 | 25 |
| DoxFumi TP53 mut | 1.30 (0.52-3.23) | 0.574 | 28 | 19 |
| Epirubicin | 1.13 (0.56-2.25) | 0.737 | 90 | 32 |
| Epirubicin no crossover | 1.32 (0.53-3.29) | 0.549 | 61 | 19 |
| Epirubucin TP53 wt | 0.93 (0.44-1.96) | 0.853 | 74 | 28 |
| Epirubicin TP53 mut | 3.97 (0.41-28.42) | 0.233 | 16 | 4 |
| Paclitaxel | 0.86 (0.48-1.54) | 0.614 | 99 | 45 |
| Paclitaxel no crossover | 0.95 (0.40-2.25) | 0.904 | 57 | 21 |
| Paclitaxel TP53 wt | 0.96 (0.49-1.91) | 0.913 | 77 | 33 |
| Paclitaxel TP53 mut | 0.51 (0.16-1.59) | 0.245 | 22 | 12 |

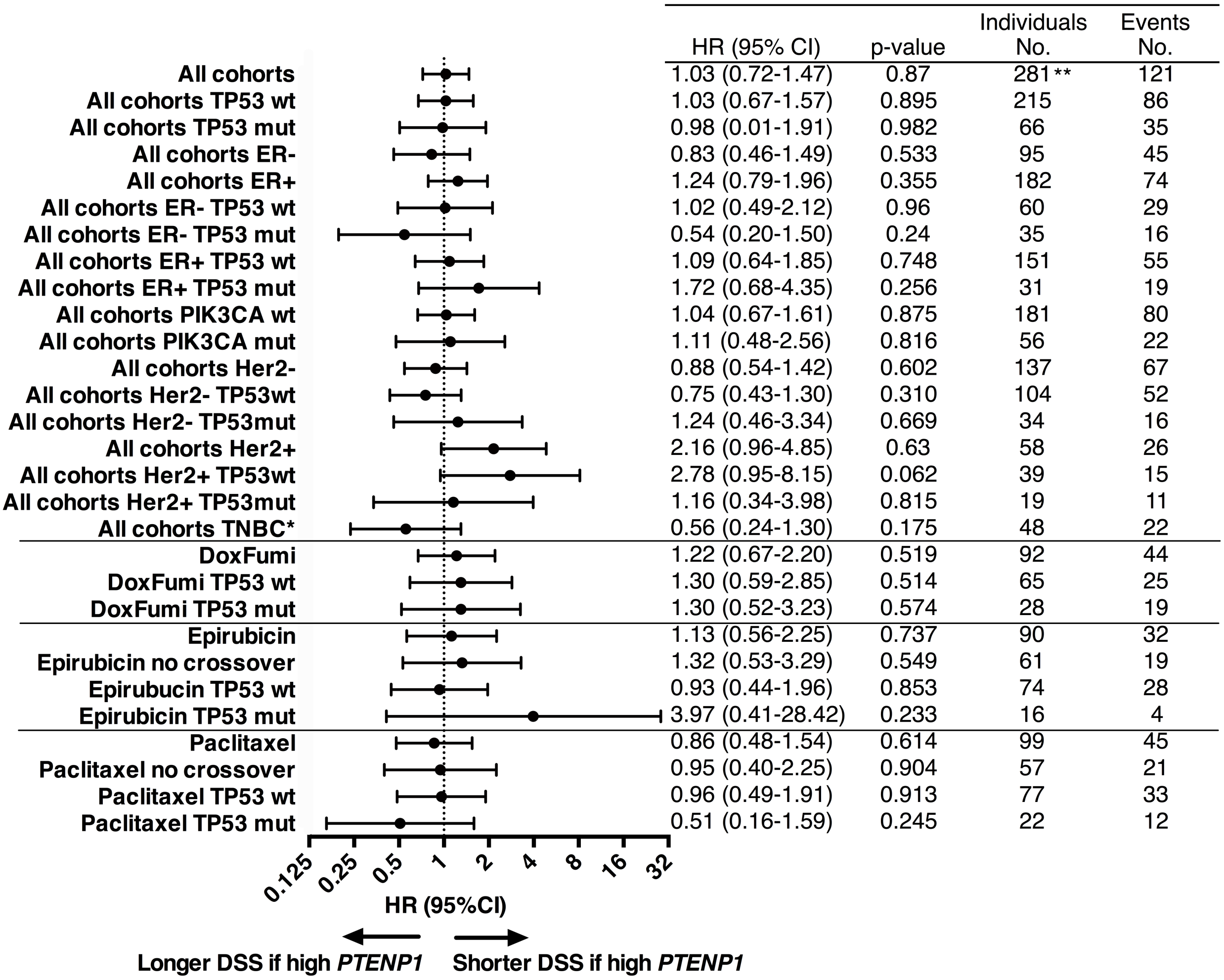

Supplement: Supplementary file 4 — Supplementary material 4 (PDF 4508 kb) [file 10549_2017_4160_MOESM4_ESM.pdf]
