# Supplementary material for: High PTEN gene expression is a negative prognostic marker in human primary breast cancers with preserved p53 function
Source: Breast Cancer Res Treat. 2017 Feb 17;163(1):177–90. doi: 10.1007/s10549-017-4160-5 (PMC5387035; doi:10.1007/s10549-017-4160-5)
Supplement: Supplementary file 5 — Supplementary material 5 (PDF 1882 kb) [file 10549_2017_4160_MOESM5_ESM.pdf]

High *PTEN* gene expression is a negative prognostic marker in human primary breast cancers with preserved p53 function

Breast Cancer Research and Treatment

Authors:

Synnøve Yndestad[1,2], Eilin Austreid[1], Stian Knappskog[1,2], Ranjan Chrisanthar[3], Peer Kåre Lilleng[4,5], Per Eystein Lønning[1,2], Hans Petter Eikesdal[1,2*]

Author details:

[1]Section of Oncology, Department of Clinical Science, University of Bergen, Bergen, Norway.

[2]Department of Oncology, Haukeland University Hospital, Bergen, Norway.

[3]Section of Molecular Pathology, Department of Pathology, Oslo University Hospital, Oslo, Norway.

[4]Department of Pathology, Haukeland University Hospital, Bergen, Norway.

[5]Laboratory of Pathology, Department of Clinical Medicine, University of Bergen, Bergen, Norway.

***Correspondence:** hans.eikesdal@k2.uib.no

Online Resource 5

a-b Forest plot for the association between tumor protein staining by immunohistochemistry (IHC) and recurrence-free (**a**) or disease-free survival (**b**) in patients with locally advanced breast cancer. Results are presented as individual hazard ratios (HRs) with corresponding 95% confidence intervals (CIs), based on tissue microarray staining and survival data from Study 3 (epirubicin/paclitaxel trial). HR>1 indicates that the survival of patients with protein staining is shorter than that of patients without protein staining in the tumors, while HR<1 indicates the opposite. Number of samples analyzed per protein vary due to technical issues during tissue microarray staining. *One case censored before the earliest event in a stratum. RFS: recurrence-free survival, DSS: disease-specific survival, wt: wildtype, mut: mutated, ER: estrogen receptor, pAkt: phosphorylated Akt (Ser 473), pS6K (Ser 371): phosphorylated S6 kinase (Ser 371), pS6K (Thr 389): phosphorylated S6 kinase (Thr 389), p4EBP1: phosphorylated 4EBP1 (Thr 70), c: cytoplasmic staining, n: nuclear staining

a

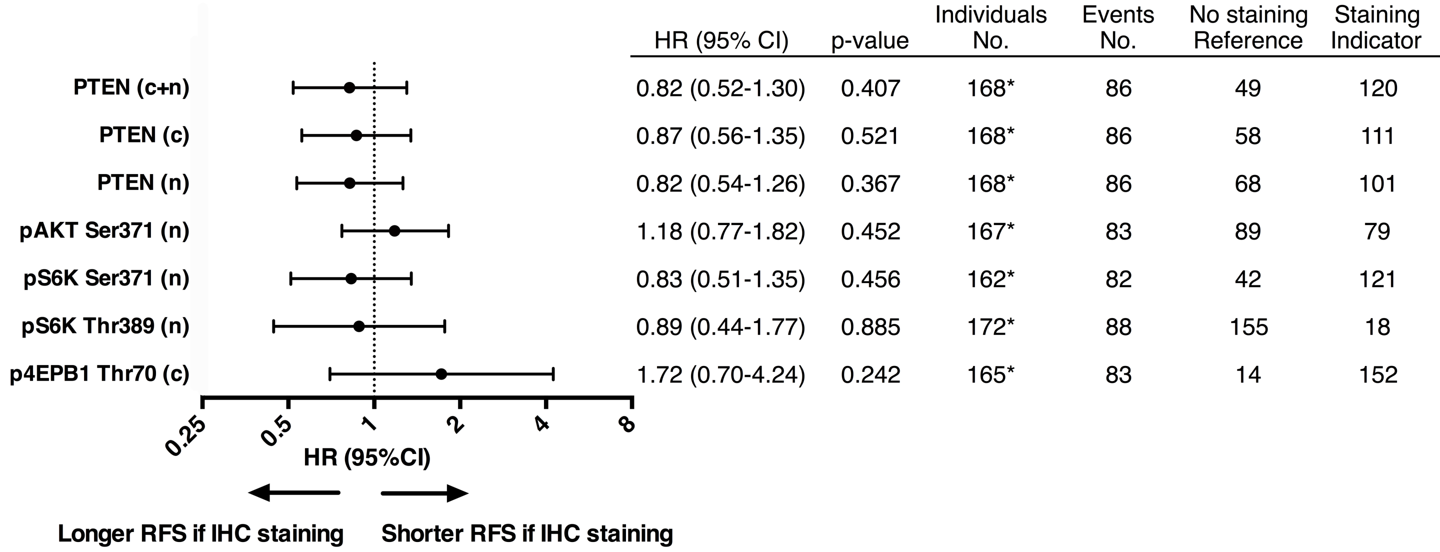

	HR (95% CI)	p-value	Individuals No.	Events No.	No staining Reference	Staining Indicator
PTEN (c+n)	0.82 (0.52-1.30)	0.407	168*	86	49	120
PTEN (c)	0.87 (0.56-1.35)	0.521	168*	86	58	111
PTEN (n)	0.82 (0.54-1.26)	0.367	168*	86	68	101
pAKT Ser371 (n)	1.18 (0.77-1.82)	0.452	167*	83	89	79
pS6K Ser371 (n)	0.83 (0.51-1.35)	0.456	162*	82	42	121
pS6K Thr389 (n)	0.89 (0.44-1.77)	0.885	172*	88	155	18
p4EPB1 Thr70 (c)	1.72 (0.70-4.24)	0.242	165*	83	14	152

b

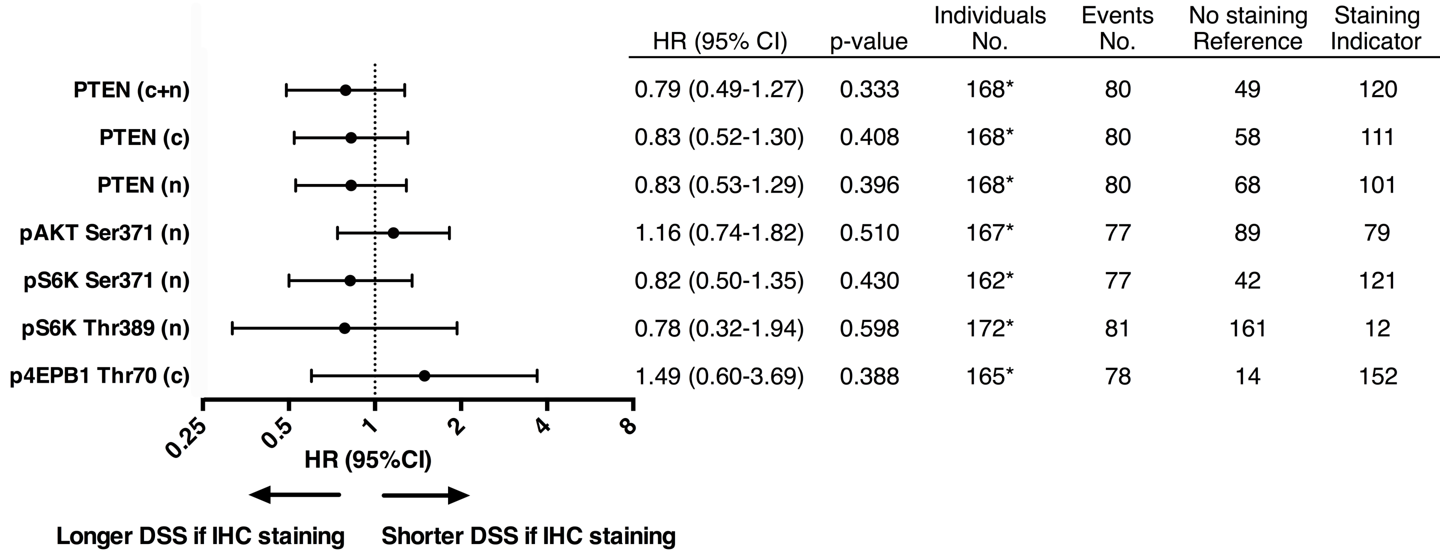

	HR (95% CI)	p-value	Individuals No.	Events No.	No staining Reference	Staining Indicator
PTEN (c+n)	0.79 (0.49-1.27)	0.333	168*	80	49	120
PTEN (c)	0.83 (0.52-1.30)	0.408	168*	80	58	111
PTEN (n)	0.83 (0.53-1.29)	0.396	168*	80	68	101
pAKT Ser371 (n)	1.16 (0.74-1.82)	0.510	167*	77	89	79
pS6K Ser371 (n)	0.82 (0.50-1.35)	0.430	162*	77	42	121
pS6K Thr389 (n)	0.78 (0.32-1.94)	0.598	172*	81	161	12
p4EPB1 Thr70 (c)	1.49 (0.60-3.69)	0.388	165*	78	14	152
